# Targeted Biomarker Discovery by High Throughput Glycosylation Profiling of Human Plasma Alpha1-Antitrypsin and Immunoglobulin A

**DOI:** 10.1371/journal.pone.0073082

**Published:** 2013-09-09

**Authors:** L. Renee Ruhaak, Carolien A. M. Koeleman, Hae-Won Uh, Jord C. Stam, Diana van Heemst, Andrea B. Maier, Jeanine J. Houwing-Duistermaat, Paul J. Hensbergen, P. Eline Slagboom, André M. Deelder, Manfred Wuhrer

**Affiliations:** 1 Center for Proteomics and Metabolomics, Leiden University Medical Center, Leiden, The Netherlands; 2 Section Medical Statistics, Department of Medical Statistics and Bioinformatics, Leiden University Medical Center, Leiden, The Netherlands; 3 Section Biomolecular Imaging, Department of Biology, Utrecht University, Utrecht, The Netherlands; 4 Department of Gerontology and Geriatrics, Leiden University Medical Center, Leiden, The Netherlands; 5 Section Molecular Epidemiology, Department of Medical Statistics and Bioinformatics, Leiden University Medical Center, Leiden, The Netherlands; 6 Netherlands Consortium of Healthy Aging, Leiden, The Netherlands; Moffitt Cancer Center, United States of America

## Abstract

Protein N-glycosylation patterns are known to show vast genetic as well as physiological and pathological variation and represent a large pool of potential biomarkers. Large-scale studies are needed for the identification and validation of biomarkers, and the analytical techniques required have recently been developed. Such methods have up to now mainly been applied to complex mixtures of glycoproteins in biofluids (e.g. plasma). Here, we analyzed N-glycosylation profiles of alpha1-antitrypsin (AAT) and immunoglobulin A (IgA) enriched fractions by 96-well microtitration plate based high-throughput immuno-affinity capturing and N-glycan analysis using multiplexed capillary gel electrophoresis with laser-induced fluorescence detection (CGE-LIF). Human plasma samples were from the Leiden Longevity Study comprising 2415 participants of different chronological and biological ages. Glycosylation patterns of AAT enriched fractions were found to be associated with chronological (calendar) age and they differed between females and males. Moreover, several glycans in the AAT enriched fraction were associated with physiological parameters marking cardiovascular and metabolic diseases. Pronounced differences were found between males and females in the glycosylation profiles of IgA enriched fractions. Our results demonstrate that large-scale immuno-affinity capturing of proteins from human plasma using a bead-based method combined with high-throughput N-glycan analysis is a powerful tool for the discovery of glycosylation-based biomarker candidates.

## Introduction

Glycosylation occurs on more than 50% of human proteins [1] and proper glycosylation is essential for the survival of most multicellular organisms. N-Glycans have important functions in several biological processes such as cellular interaction, differentiation, and immunological defense mechanisms [1]–[6]. Protein N-glycosylation is very diverse, and more than 100 different N-glycans may be found on a specific protein [7]. The glycan signature, i.e. the total plasma N-glycome of an individual, reflects both genetic and physiological factors [8] and is highly reproducible in a given physiological state [9], [10]. However, when the physiological state changes, e.g. due to aging or disease, the glycan pattern can change dramatically [9]. Due to the large variability of protein glycosylation and its reflection of physiological and pathological conditions, protein glycosylation patterns have been marked as a large field of potential biomarkers [11].

To identify and validate biomarkers, large-scale studies are needed, and the analytical methods required for the evaluation of protein glycosylation patterns at the glycan level in larger sample sets have only recently been developed (e.g. [12]–[14]). Up to now, these methods have mainly been applied for the evaluation of plasma N-glycosylation profiles (e.g. [8], [15]–[17]). However, some constraints are associated with the interpretation of the results from such analyses. As the profiles originate from the total protein pool present in plasma, changes in the N-glycosylation profile may be caused by alterations in protein concentration, or by changes in protein-specific glycosylation patterns. Another aspect is that glycans from high abundant proteins dominate the glycan pattern, and changes in the glycosylation signature of less abundant proteins will not be detectable.

There is, therefore, a need for fast, large-scale glycan analysis of specific glycoproteins or groups of glycoproteins. Current methods for glycoprotein enrichment often comprise lectins or (immuno-)affinity purification (e.g. [18], [19]). Except for affinity capturing of IgG [20]–[22], however, this has to our knowledge not been applied in large-scale studies. Whilst the IgG glycosylation profiling studies applied Protein A and/or Protein G purification, we used capturing using immobilized antibodies which is the method of choice for the isolation of various proteins from serum and plasma.

Human plasma contains a large variety of proteins at a wide range of concentrations [23]. To evaluate the feasibility of large-scale immunocapturing and subsequent N-glycan analysis, we decided to analyze N-glycans of two proteins occurring in the circulation at high abundance: alpha-1-antitrypsin (AAT), a protease inhibitor which is mainly produced by hepatocytes [24], and immunoglobulin A (IgA), after IgG the second most abundant antibody class in plasma, which is produced in the B-cells in the bone marrow [25]. It is expected that the glycosylation of these two proteins is regulated differently, given their different origins, and it may be speculated that these 2 proteins reflect glycosylation profiles of their sites of origin.

Human IgA exists in two subclasses; IgA1 and IgA2. Plasma IgA consists for 90% of IgA1, and 10% IgA2, while secretory IgA may contain up to 50% IgA2 [25]. IgA1 carries two N-glycosylation sites, while up to 5 N-glycans may be attached to IgA2 [26]. O-glycans have also been observed on IgA [27], [28]. IgA1 has been reported to carry mostly biantennary and triantennary glycans, which are often decorated with sialic acid, fucose and/or bisecting GlcNAc [29], [30].

Alpha-1-antitrypsin is normally present in plasma at a concentration of 0.88–1.64 mg/ml in healthy individuals [31], but since AAT is an acute-phase reactant, its concentration may increase 3–4 fold upon infection. The protein carries 3 N-glycosylation sites, which mainly carry bi- and tri- antennary glycans [32]–[34]. Decreased levels of serum AAT are associated with increased incidence of chronic obstructive pulmonary disease (COPD), and liver malfunction [24].

Large-scale analysis of glycans from single glycoproteins or mixtures of a few glycoproteins may be performed at the glycopeptide level or at the glycan level. Using glycopeptide analysis, site-specific glycosylation profiles may be obtained [21]. However, analysis at the glycan level may also be advantageous as such an approach is universal, while analytical procedures at the glycopeptide level have to be optimized for each glycoprotein.

We here describe an approach for high-throughput immuno-affinity capturing in combination with N-glycan analysis for targeted glycan based biomarker discovery. To this end, both commercially available antibody coated beads (anti-immunoglobulin A, IgA), as well as beads that were coated in-house with VHH-antibodies (small, camelid-derived single domain antibody fragments, anti-alpha1-antitrypsin, AAT) were used. We have previously developed a workflow for high-throughput analysis of N-glycans from plasma using multiplexed capillary gel electrophoresis with laser induced fluorescence detection (CGE-LIF) [35]. Here, we apply this method for profiling of AAT- and IgA-associated glycosylation patterns of 2415 samples of the Leiden Longevity Study (LLS). This is a cohort which consists of long-lived siblings together with their offspring and the partners thereof, and is aimed at the identification of markers for healthy aging [36]. While changes in glycosylation of the IgA enriched fraction were predominantly determined by the sex of the individual, AAT glycosylation patterns could be associated with age and sex, as well as with several parameters reflecting metabolic regulation and cardiovascular as well as inflammatory disease states (BMI, cholesterol, HDL, triglyceride, glucose, insulin and CRP levels).

## Experimental Section

### Materials

Dimethylsulfoxide (DMSO), Nonidet P-40 (NP-40), formic acid, triethylamine (TEA), aminopyrene-1,3,6-trisulfonic acid (APTS), 2-picoline borane, trifluoroacetic acid (TFA), and agarose-linked goat anti-human IgA antibody were obtained from Sigma-Aldrich (Zwijndrecht, The Netherlands). Sodium dodecyl sulfate (SDS) was bought from United States Biochemicals (Cleveland, OH). PNGase F was obtained from Roche Diagnostics (Mannheim, Germany). Biogel P-10 was obtained from Bio-Rad (Veenendaal, The Netherlands) and citric acid from Merck (Darmstadt, Germany). Acetonitrile (ACN) was purchased from Biosolve (Valkenswaard, The Netherlands). 0.45 μm GHP filter plates were obtained from Pall Corporation (Ann Arbor, MI) and 96-well V-bottom deep well plates from Westburg (Leusden, The Netherlands). PCR plates for measurement in the DNA-sequencer were obtained from Thermo Fischer Scientific via Westburg (Leusden, The Netherlands). 96-well polypropylene filter plates containing a 10 μm PE frit were obtained from Orochem (Lombard, IL). Milli Q water was used throughout (Merck Millipore). Standard plasma for the repeatability study and plate-standards was obtained from a healthy donor.

### Participants of the Leiden Longevity Study

In the Leiden Longevity Study (LLS), Caucasian families were recruited if at least two long-lived siblings were alive and fulfilled the age-criterion of 89 years or older for males and 91 year or older for females, representing less than 0.1% of the Dutch population in 2001. As no proper controls exist for this age group, for further studies the offspring of these long-lived nonagenarians were included with their partners as controls. The offspring carry on average 50% of the genetic propensity of their long-lived parent and were shown to have a 30% lower mortality rate compared with their birth cohort [36]. Their similarly aged partners, with whom most have had a relationship for decades, were included as age- and environmentally matched controls. In total, 943 long-lived siblings were included, 1671 offspring with a mean age of 59.4 and 744 partners with a mean age of 58.7 years [36]. For the offspring of long-lived siblings and their partners, non-fasting serum samples were taken at baseline for the determination of endocrine and metabolic parameters. Additional information was collected from the generation of offspring and controls, including self-reported information on height and weight and information on medical history from the participants' treating physicians (for details see Table 1 of [15]).

**Table 1:** Peak annotation of AAT-enriched glycans.

*Fraction*	*Composition*	*Spiking*	*Literature*	*PGC-LC-ESI-IT-MS*
AAT-2	Hex6HexNAc5NeuAc3	X	[33], [34]	960.8 [M+3H]^3+^*
AAT-3	Hex5HexNAc4NeuAc2	X	[33], [34]	1111.9 [M+2H]^2+^
AAT-4	Hex5HexNAc5NeuAc2	X		
AAT-5	Hex5HexNAc4Fuc1NeuAc2	X	[33], [34]	1184.9 [M+2H]^2+^
AAT-6	Hex5HexNAc5Fuc1NeuAc2	X		
AAT-7	Hex7HexNAc6Fuc1NeuAc3	X	[33]	
AAT-8	Hex6HexNAc5NeuAc2	X	[33]	
AAT-9	Hex6HexNAc5NeuAc2	X	[33]	
AAT-10	Hex6HexNAc5Fuc1NeuAc2	X	[33]	
AAT-11	Hex6HexNAc5Fuc1NeuAc2	X	[33]	
AAT-12	Hex5HexNAc4NeuAc1	X	[33], [34]	966.4 [M+2H]^2+^
AAT-13	Hex5HexNAc4NeuAc1	X	[33], [34]	966.4 [M+2H]^2+^
AAT-14	Hex5HexNAc4Fuc1NeuAc1	X	[33]	1039.4 [M+2H]^2+^
AAT-15	Hex5HexNAc5Fuc1NeuAc1	X	[33]	1140.9 [M+2H]^2+^
AAT-16	Hex6HexNAc5NeuAc1	X		
AAT-17	Hex3HexNAc4Fuc1	X		
AAT-20	Hex4HexNAc4	X		
AAT-21	Hex5HexNAc4	X		
AAT-22	Hex5HexNAc4Fuc1	X		893.9 [M+2H]^2+^

Glycan compositions were annotated based on spiking experiments with human plasma and on literature. Compositions are given in terms of Hex: hexose, HexNAc: N-acetylhexosamine, Fuc: fucose, NeuAc: N-acetylneuraminic acid. *, observed by RP-LC-MS.

### Ethics statement

The study protocol was approved by the Leiden University Medical Center ethical committee and an informed consent was signed by all participants prior to participation in the study.

### Phenotypic parameters

All serum measurements were performed with fully automated procedures. For insulin, the Immulite 2500 from DPC (Los Angeles, CA, USA) was applied. For glucose, hsCRP, total cholesterol, HDL-cholesterol (HDL-C) and triglycerides the Hitachi Modular or the Cobas Integra 800, both from Roche, Almere, The Netherlands were applied. LDL-cholesterol level (LDL-C) was calculated using the Friedewald formula (LDL-C  =  total cholesterol – HDL-C – (triglycerides/2.2); unit mmol/L) and set to missing if plasma triglyceride concentration exceeded 4.52 mmol/L.

### Preparation of anti-AAT beads

The anti-human AAT llama antibody fragments (VHHs) were kindly provided by BAC (Leiden, The Netherlands). VHHs were purified, dialyzed against PBS and coupled to NHS-activated Sepharose 4 fast flow beads (GE Healthcare) according to the manufacturer's instructions, at 3 mg/mL overnight. Using SDS-PAGE with Coomassie staining it was confirmed that 80% of the VHHs were coupled to the beads. Beads were stored in 20% ethanol in water at 4°C.

### Immuno-affinity enrichment of AAT and IgA

Antibody coated beads were washed three times with 10 volumes of PBS. Six µL of anti-AAT or 20 μL of anti-IgA beads were applied per well of a 96-well filter plate. The volume was brought to 200 μL with PBS, and 10 μL of plasma were applied per well. To each 96-well plate, 2 control samples, 92 study samples and two blancs were applied. The plate was sealed with tape and incubated on a shaker for 1 h. The beads were washed with 3×200 μL PBS by vacuum filtration. After subsequent washing with 2×200 μL water, enriched glycoproteins were eluted with 100 μL of 100 mM formic acid into a V-bottom microtitration plate. Samples were dried by vacuum centrifugation. To allow reuse of the antibody coated beads, beads were washed in the filter plates using 3×100 μL of 100 mM formic acid, followed by equilibration using 3×200 μL PBS. 200 μL of PBS were added to the well prior to application of the following batch of plasma samples.

### Proteomic analysis of anti-AAT and anti-IgA captured proteins

From three different subjects out of the total study cohort, anti-AAT and anti-IgA captured proteins were dried by vacuum centrifugation, dissolved in 25 µL of 25 mM NH_4_HCO_3_ and analyzed for their protein constituents using two different approaches.

For the first approach, 10 µL of the reconstituted samples were used for in-solution trypsin digestion. For this purpose, proteins were first reduced and alkylated using dithiothreitol (5 mM in 25 mM NH_4_HCO_3_, 30 min, 60°C) followed by iodoacetamide (15 mM in 25 mM NH_4_HCO_3_, 30 min, RT, in the dark). The alkylation reaction was stopped by adding 15 mM of dithiothreitol. Then, 50 ng trypsin (sequencing grade modified Trypsin, Promega, Madison, WI) was added and proteins were digested overnight at 37°C. The digestion reaction was stopped using 2 µL of 5% TFA.

The digests were then analyzed by LC-MS using a Ultimate 3000 RSLCnano LC system (Thermo Scientific, Sunnyvale, CA) coupled to a HCTultra ion trap mass spectrometer (Bruker Daltonics, Bremen, Germany). The samples were injected onto an Acclaim C_18_ PepMap100 trapping column (100 µm×2 cm, 5 µm, 100 Å (Thermo Scientific) and washed with 100% A (3% ACN in 0.1% formic acid) at 5 µL/min for 6 min. Following valve switching, peptides were separated on an Acclaim C_18_ PepMapRSLC column (75 µm×150 mm, 2 µm, 100 Å) at a constant flow of 300 nL/min. The peptide elution gradient was from 3 to 40% B (95% ACN in 0.1% formic acid) in 48 min followed by an increase to 65% B in 10 min. The nanoflow LC was coupled to the mass spectrometer using a nano-electrospray ionization source. The spray voltage was set at 1.2 kV and the temperature of the heated capillary was set to 165°C. Eluting peptides were analyzed using the data dependent MS/MS mode over a 300–1500 *m/z* range. The five most abundant ions in an MS spectrum were selected for MS/MS analysis by collision-induced dissociation using helium as the collision gas.

Peak lists were generated using DataAnalysis 4.0 software (Bruker Daltonics) and exported as Mascot Generic (MGF) files. These files were searched against the human Swissprot database (Date of release 21-05-2012, 536029 sequences, 20319 *Homo sapiens*) with the Mascot search algorithm (Mascot 2.2, Matrix Science, London, UK) using Mascot Deamon. An MS tolerance of 0.6 Da (with #^13^C = 1) and a MS/MS tolerance of 0.5 Da was used. Trypsin was designated as the enzyme and up to one missed cleavage site was allowed. Carbamidomethylcysteine was selected as a fixed modification and oxidation of methionine as a variable modification. Only significant protein hits with at least two unique peptides with a score above 30 were selected.

For the second approach, anti-AAT and anti-IgA captured proteins were analyzed by SDS-PAGE using a pre-cast gradient gel (Invitrogen NuPAGE 4–12% Bis-Tris Gel) and proteins were visualized using Colloidal Blue Staining (Life Technologies). The major protein band from the anti-AAT and anti-IgA samples was selected for in-gel digestion. For this purpose, bands were cut into small pieces, and washed with 25 mM NH_4_HCO_3_ followed by dehydration with ACN for 10 min. For reduction and alkylation, dried gel particles were first incubated with 10 mM dithiothreitol for 30 min at 56°C. Following dehydration with ACN, gel plugs were subsequently incubated in 55 mM iodoacetamide for 20 min at room temperature. After two rounds of washing with 25 mM NH_4_HCO_3_ and dehydration with ACN, the gel particles were completely dried by vacuum centrifugation. The dried gel particles were re-swollen for 15 min on ice after the addition of 15 μL of a trypsin solution (5 ng/μl in 25 mM NH_4_HCO_3_). Following this, 20 µL of 25 mM NH_4_HCO_3_ was added and the samples were kept on ice for an additional 30 min. Tryptic digestion was subsequently performed overnight at 37°C. The overlaying digestion-solution containing the tryptic peptides was collected (extract 1). One additional round of extraction with 20 μl 0.1% TFA was used to extract peptides from the gel plugs and this extract was pooled with extract 1.

MALDI-ToF-MS analyses were performed on an Ultraflex II mass spectrometer (Bruker Daltonics) using 2,5-dihydroxybenzoic acid (5 mg/mL of 50% ACN/0.1% trifluoroacetic acid) as a matrix. The mass spectrometer was used in the positive ion reflector ion mode. Spectra were imported in Flexanalysis 3.0 (Bruker Daltonics) for smoothing, baseline subtraction and peak picking. Peak lists were searched against the human Swissprot database using the Mascot search algorithm in Biotools (version 3.2, build 3.34, Bruker Daltonics). Trypsin was selected as the enzyme and one missed cleavage was allowed. Carbamidomethylcysteine was selected as a fixed modification and oxidation of methionine as a variable modification. The MS tolerance was set at 100 ppm.

### Glycan release, labeling and purification

N-glycans were released from the enriched glycoprotein samples similarly to the procedure described previously for plasma [14] with slight modifications. Shortly, proteins were denatured after resuspension in 2 μL 2% SDS by incubation at 60°C for 10 min. Subsequently, 2 μL 2% NP-40 containing 0.2 mU of PNGase F was added to the samples. The samples were incubated overnight at 37°C for N-glycan release. Labeling of oligosaccharides was performed as published [35], [37] with slight modifications: 2 μL of a freshly prepared solution of label (APTS; 20 mM in 3.6 M citric acid) and 2 μL of freshly prepared reducing agent solution (0.2 M 2-picoline borane in DMSO, see [38]) were added, the plate was sealed using adhesive tape and after 5 min of shaking, the samples were incubated at 37°C for 16 h.

Hydrophilic interaction liquid chromatography (HILIC)-SPE was applied to purify labeled N-glycans. An amount of 100 μL of a 100 mg/mL suspension of Biogel P-10 in water/ethanol/ACN (70∶20∶10, v/v) was applied to each well of a 0.45 μm GHP filter plate (Pall Corporation, Ann Arbor, MI). Solvent was removed by application of vacuum using a vacuum manifold (Millipore, Billerica, MA). All wells were prewashed using 5×200 μL water, followed by equilibration using 3×200 μL ACN/water (80∶20, v/v). The samples were loaded to the wells, and the plate was shaken for 5 min. The wells were subsequently washed using 5×200 μL ACN/100 mM triethylamine (TEA) adjusted to pH 8.5 with acetic acid (80∶20, v/v), followed by 3×200 μL ACN/water (80∶20, v/v). Washing steps were performed by addition of solutions, incubation for 30 s, and removal of solvent by vacuum. Water (100 μL) was applied followed by 5 min incubation to allow swelling of the Biogel P-10 particles. For elution, samples were incubated for 5 min with 200 μL water prior to collection of eluates by vacuum in a 96-well V-bottom polypropylene deep well plate. The elution step was repeated, and the 2×200 μL eluates were combined and analyzed immediately by CGE-LIF or stored at −20°C until usage.

### CGE-LIF analysis

Multiplexed CGE-LIF analysis was performed as previously described [35]. Shortly, 2 μL of N-glycan eluate were added to 60 μL of DMSO in a PCR plate (Thermo Fischer Scientific, Westburg, Leusden, The Netherlands). Plates were sealed and turned several times for thorough mixing and subsequently centrifuged prior to analysis using an ABI-3730 DNA sequencer (Applied Biosystems). The injection voltage was set at 7.5 kV, while the running voltage was 10 kV. The system was equipped with a 48-channel array with capillaries of 50 cm, and the capillaries were filled with POP-7 buffer (Applied Biosystems). The 3730 running buffer was obtained from Applied Biosystems. Data were collected with a frequency of 10 Hz for 50 min using ABI Data Collection software v.2.0.

### Data processing

Data files were converted to xml files using DataFileConverter, which is supplied by Applied Biosystems. Files were then loaded into Matlab software (version 2007a; The Mathworks, Inc., Natick, MA), and after smoothing, the data were cropped to 17000 data points to reduce the data volume. Alignment using Correlation optimized warping (COW) was performed as reported previously [39], [40] using a representative electropherogram as the reference file. Segment length and slack size were optimized according to [39]. After smoothing and background adjustment, the peak integrals were determined (22 for AAT and 15 for IgA). Peak integrals were normalized on the signal intensity of Peak 3 (AAT) or Peak 5 (IgA). Peak AAT-3 (annotation Hex_5_HexNAC_4_NeuAc_2_) was chosen as it was of highest abundance and therefore consistently identified in all of the samples. Similarly, peak IgA-5 (annotation Hex_5_HexNAc_4_Fuc_1_NeuAc_2_) was chosen being among the major signals.

For the determination of the intra-batch variation 4 standard plasma samples, obtained from a healthy donor, were analyzed. Glycoproteins were enriched and N-glycans were released, labeled, purified, and subsequently analyzed using the DNA sequencer in parallel on one plate. The procedure was repeated on four consecutive days. Per day averages as well as standard deviations were calculated from the four samples. RSDs were determined and the average RSDs from the four consecutive days were calculated per peak. To determine inter-batch repeatability, four standard samples were analyzed. N-glycans were released, labeled, purified, and subsequently analyzed using the DNA sequencer. The procedure was repeated on four consecutive days. For each sample position the averages and standard deviations over the four days were calculated. RSDs and subsequently average RSDs over all four samples were calculated.

### Statistics

The samples of 2415 participants were divided over 28 individual plates to analyze AAT- and IgA-associated N-glycosylation patterns. To correct for batch effects, the N-glycosylation values were regressed on the categorical variable batch memberships. The standardized residuals of this model were used for further statistical analysis.

Since the LLS comprises multiple participants from the same family, robust standard errors were obtained. P-values<0.05 were regarded statistically significant. First, linear regression was used to explore the relationships between each of the response variables and covariates – age and sex – adjusting for the family status (offspring or partner of the offspring). Then, linear regression was used to explore relationships between N-glycosylation features and covariates – BMI (ln-transformed), levels of cholesterol, HDL-cholesterol, LDL-cholesterol, triglyceride (ln transformed) and glucose as well as insulin level (ln transformed) – adjusting for the family status (offspring or partner of the offspring) age, sex and the age × sex interaction.

To determine potential biomarkers for longevity, logistic regression was applied to investigate whether glycosylation feature (independent variable) was predictive in classifying the family status after adjustment for age, sex, and their interaction. In that respect, the response variable (the family status) is coded as 0 ( =  partner) and 1 ( =  offspring of long-lived sibling). Finally, linear regression was used to explore relationships between glycosylation features and disease status – levels of CRP and prevalence of MI, cardiovascular diseases or diabetes- adjusting for age, sex and their interaction.

Analyses were performed using STATA 10 (StataCorp LP, College Station, Texas, USA) and R version 2.9.0 (R Development Core Team).

## Results

### Protein enrichment

Glycoproteins were enriched from human plasma using high-throughput immuno-affinity capturing in a 96-well format. Anti-AAT and anti-IgA antibody coated beads were applied to each of the wells of a filter plate, after which plasma was added to the wells. After incubation for 1 h and thorough washing with PBS and water, the enriched glycoprotein fraction was eluted using 100 mM formic acid.

To check the purity of the enriched glycoprotein samples, we performed a proteomic analysis of the samples from three different subjects from our study cohort. First, we analyzed these samples by SDS-PAGE (Fig. SF1 in Supporting Information S1) and selected the major bands for in-gel digestion using trypsin. The digests were subsequently analyzed by MALDI-ToF-MS and the resulting peptide mass fingerprints were searched against the human Swissprot database. As expected, this resulted in the identification of AAT and IgA1 (heavy chain), respectively and the major ion species in the MS spectra of all three subjects corresponded to either of these proteins (Fig. SF2 in Supporting Information S1).

In addition, we performed an in-solution tryptic digestion of the total pool of immunocaptured proteins followed by nanoLC-ion trap-MS analysis (Tables ST1 and ST2 in Supporting Information S1). Again, this resulted in the largest number of peptides identified for IgA1 (heavy chain) and AAT in the corresponding samples. Several co-purified proteins were observed within the AAT sample but based on the band intensities in SDS-PAGE (Fig. SF1 in Supporting Information S1), total number of peptides identified and known N-glycosylation of some of them, the contribution to the downstream glycan analysis of these samples was expected to be small.

After IgA1 (heavy chain) and serum albumin, the largest set of peptides identified in the LC-MS analysis of the tryptic digest of the IgA enriched samples corresponded to the Ig kappa and lambda light chains (Table ST1 in Supporting Information S1). They are also visible in the lower region on the SDS-PAGE gel of these samples (Fig. SF1 in Supporting Information S1). In two of the samples (from subject 2 and 3) we also identified IgA2 which is known to be the minor IgA species within the circulation [25]. In one of the samples (subject 1), 2 peptides from IgG were observed indicating trace amounts of IgG, which will probably not affect the results from our IgA glycan profiling analysis.

### Glycan peak annotation

**Figure 1:**
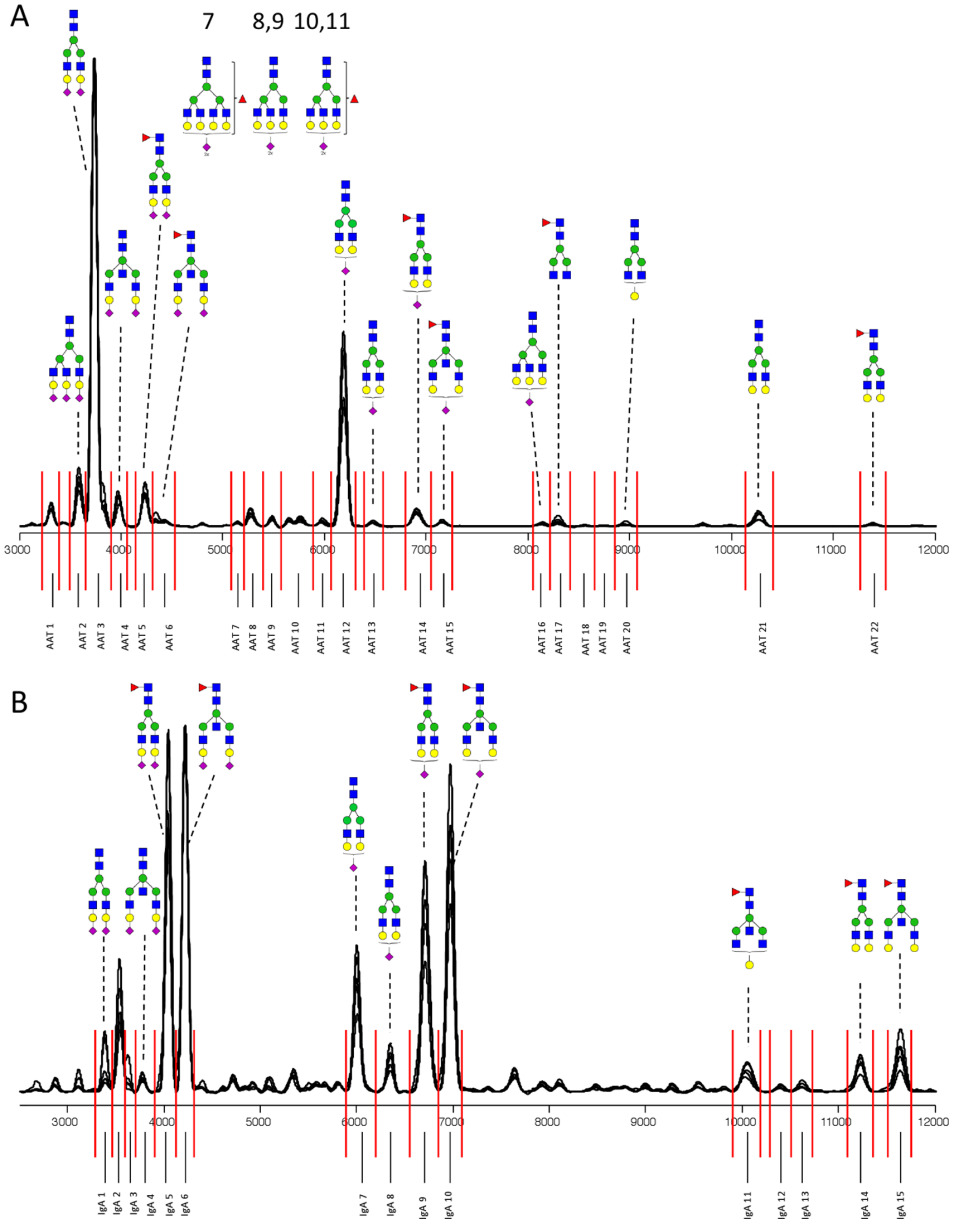
Representative pool of electropherograms from enriched glycans randomly chosen from the LLS samples. Electropherograms from the AAT-enriched fraction are depicted in (A), while electropherograms from the IgA-enriched fraction are depicted in (B). Compositions and structural schemes are given in terms of N-acetylglucosamine (blue square), mannose (green circle), galactose (yellow circle), N-acetylneuraminic acid (purple diamond) and fucose (red triangle).

We recently developed a platform for high-throughput analysis of APTS-labeled glycans using a multiplexed CGE-LIF instrument [35]. Using this platform, 96 samples can be measured within 3 hours, which makes it highly suitable for large-scale studies. Previously, we obtained annotations of 35 abundant plasma N-glycan structures in CGE-LIF by employing glycan standards whenever available and elucidating additional structures by HILIC-HPLC and MALDI-Fourier transform ion cyclotron resonance-MS. This previous annotation of plasma N-glycans, together with literature, was now used to annotate the observed APTS-labeled glycans in the AAT- and IgA-enriched samples. An overview of the annotation is given in Table 1 (AAT) and Table 2 (IgA). An overlay of ten annotated electropherograms that were randomly chosen from the LLS samples is depicted in Figure 1.

**Table 2:** Peak annotation of IgA-enriched glycans.

*Fraction*	*Composition*	*Spiking*	*Literature*	*PGC-LC-ESI-IT-MS*
IgA-1	Hex5HexNAc4NeuAc2	X	[29], [30]	1111.9 [M+2H]^2+^
IgA-4	Hex5HexNAc5NeuAc2	X		
IgA-5	Hex5HexNAc4Fuc1NeuAc2	X	[29], [30]	1184.9 [M+2H]^2+^
IgA-6	Hex5HexNAc5Fuc1NeuAc2	X	[29], [30]	1286.4 [M+2H]^2+^
IgA-7	Hex5HexNAc4NeuAc1	X	[29], [30]	966.4 [M+2H]^2+^
IgA-8	Hex5HexNAc4NeuAc1	X	[29], [30]	966.4 [M+2H]^2+^
IgA-9	Hex5HexNAc4Fuc1NeuAc1	X	[29], [30]	1039.4 [M+2H]^2+^
IgA-10	Hex5HexNAc5Fuc1NeuAc1	X	[29], [30]	1140.9 [M+2H]^2+^
IgA-11	Hex4HexNAc5Fuc1	X		
IgA-14	Hex5HexNAc4Fuc1	X		
IgA-15	Hex5HexNAc5Fuc1	X		

Glycan compositions were annotated based on spiking experiments with human plasma and literature. Compositions are given in terms of Hex: hexose, HexNAc: N-acetylhexosamine, Fuc: fucose, NeuAc: N-acetylneuraminic acid.

### Repeatability of the method

**Figure 2:**
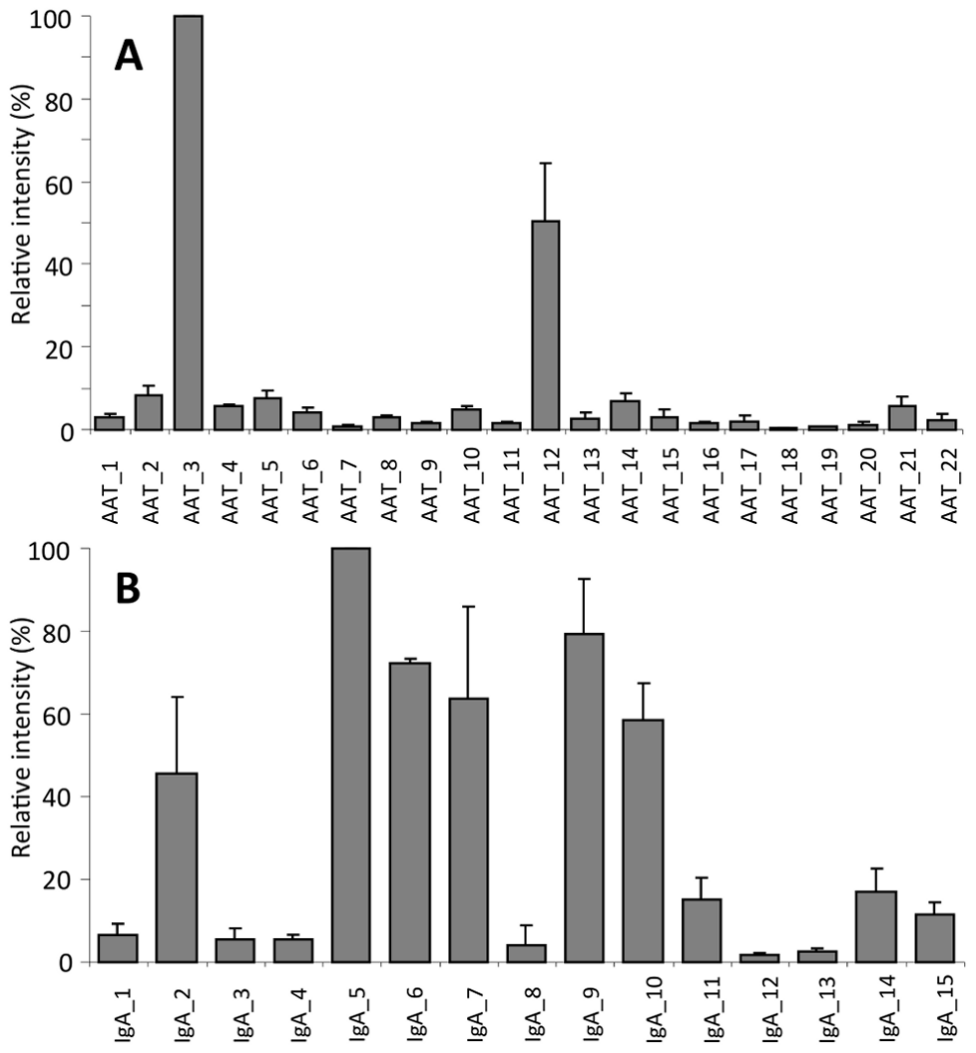
Repeatability of the enrichment procedure with subsequent analysis. Relative intensities together with their inter-batch standard deviations are depicted for AAT (A) and IgA (B).

To assess the repeatability of the procedure for large-scale protein enrichment and subsequent glycan analysis, the intra-batch as well as the inter-batch variations were calculated. The intra-batch relative standard deviation (RSD) for the AAT enriched fraction averaged over the 21 peaks was found to be 15.8% (peak 3 not included as it was used for normalization). The intra-batch RSD for the IgA enriched fraction averaged over 14 peaks was found to be 20.2% (peak 5 not included as it was used for normalization). The inter-batch standard deviation was determined by comparing the results from the four experiments, and is depicted in Figure 2. The average inter-batch RSD for the 21 peaks observed in the AAT enriched fraction was found to be 33.8%. The average inter-batch RSD for the 14 peaks in the IgA enriched fraction was found to be 32.1%.

### Relation of protein glycosylation to chronological age and sex

**Figure 3:**
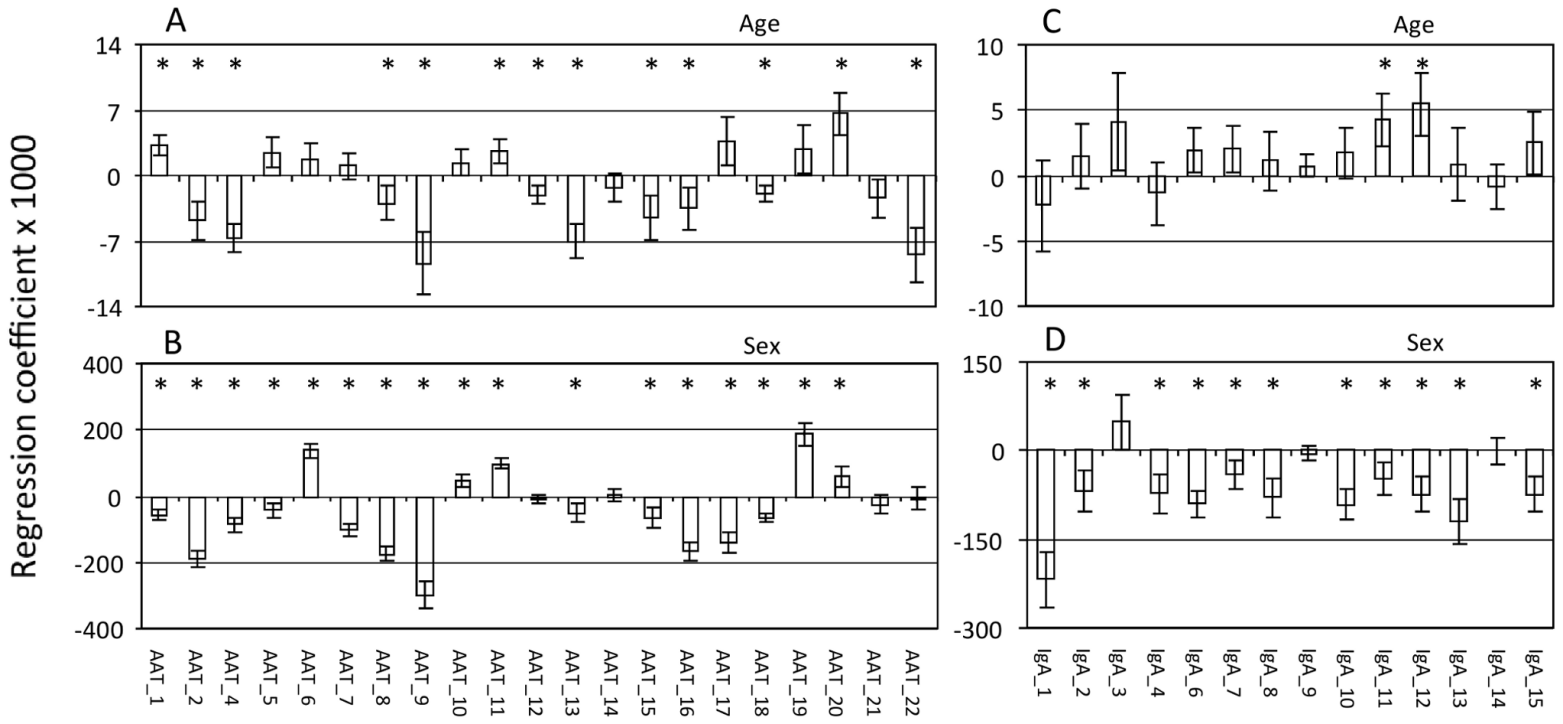
N-glycans on AAT and IgA are related to age and sex. Regression coefficients of the linear regression analysis are plotted for age (A and C) and sex (B and D) for N-glycans from AAT (A and B) and IgA (C and D). Error bars indicate standard error and significance is indicated with an asterisk.

Glycosylation profiles of the AAT enriched fraction could be obtained from 2271 individuals in the LLS, while profiles from the IgA enriched fraction were obtained from 2382 individuals. The participants in the LLS cover a large age-range (30–80 years). After batch-correction, the effects of age and sex were evaluated in both datasets (see Fig. 3, Tables ST3 and ST4 in Supporting Information S1). Several effects can be observed: non-fucosylated triantennary structures in the AAT enriched fraction (AAT-2, AAT-8, AAT-9 and AAT-16) are all negatively correlated to chronological age. Moreover, levels of most glycan features found in the IgA enriched fraction are less abundant in males than in females. Previous studies have reported that chronological age-associated changes in plasma glycosylation are usually more profound in females than in males [16], [21], [41]. Therefore, we investigated whether the interaction between chronological age and sex influences the glycosylation patterns of the AAT and IgA enriched fractions, but no significant interaction was found (data not shown).

### Protein glycosylation is affected by physiological parameters

**Figure 4:**
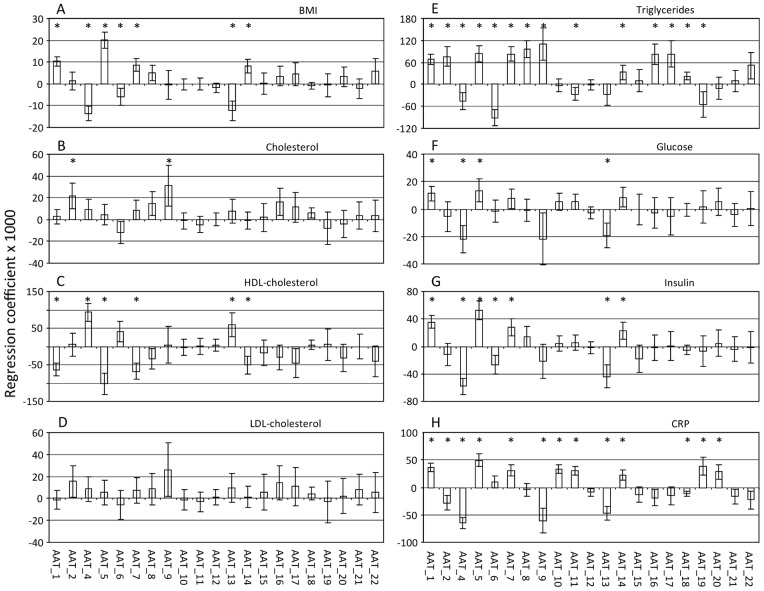
N-glycans on AAT are associated with physiological parameters. Regression coefficients of the linear regression analysis are plotted for N-glycans from AAT for BMI (A), cholesterol (B), HDL-cholesterol (C), LDL-cholesterol (D), triglycerides (E), glucose (F), insulin level (G) and C-reactive protein (H). Error bars indicate standard error and significance is indicated with an asterisk. No associations were obtained for Variable AAT-3, since the data was normalized to an individuals' level of this peak.

In two recent studies, plasma glycosylation patterns were found to be associated with altered lipid status and changes in BMI and glucose homeostasis [15], [16]. We evaluated whether glycosylation features of the AAT and IgA enriched fractions were associated with BMI, plasma levels of cholesterol, HDL-cholesterol, LDL-cholesterol, triglycerides, glucose and insulin activity. While no associations could be observed in the IgA enriched fraction (data not shown), levels of HDL-cholesterol, BMI, insulin levels, and especially levels of triglycerides are associated with several glycan features observed in the AAT enriched fractions (see Fig. 4 and Table ST5 in Supporting Information S1). This immediately raises the question whether the changes in the glycosylation profile of the AAT enriched fraction may be associated with health or disease states in general and, therefore, could be linked to longevity.

### Relation of protein glycosylation with familial longevity

The Leiden Longevity study was originally designed to study biological mechanisms that influence human life span. Here we evaluated whether glycosylation patterns of AAT and/or IgA enriched fractions are associated with familial longevity. Using a logistic regression model, none of the glycosylation features showed a significant difference between the group of offspring and the group of partners (data not shown). In addition, due to the association of various glycosylation features in the IgA- and AAT enriched fractions with sex we tested for association with familial longevity after sex stratification. Again, no significant differences were found between offspring and partners (data not shown).

### Altered protein glycosylation is associated with disease

While the glycosylation features could not be linked to familial longevity, associations were found between glycosylation patterns of the AAT enriched fractions and several physiological parameters (Fig. 4 and Table ST5 in Supporting Information S1). This raised the question whether glycans in the AAT enriched fractions may reflect specific disease states. High levels of plasma C-reactive protein (CRP) indicate an acute inflammatory response, and may be regarded as a marker for the presence of inflammatory diseases [42]. Moreover, plasma N-glycosylation was previously reported to be correlated to CRP levels [43]. Therefore, we assessed the relation between CRP levels and glycosylation features of the AAT enriched fractions. The results are depicted in Figure 4 and Table ST6 in Supporting Information S1. A large number of glycosylation features were found to be associated with CRP levels with high significance, indicating that the glycosylation profiles of AAT enriched fractions are indeed altered in diseased individuals showing systemic inflammation.

## Discussion

We here present the first study on large scale protein enrichment using immuno-affinity capturing with subsequent high-throughput N-glycan analysis. In the present study we have chosen to use a bead-based enrichment strategy, using commercially available anti-IgA antibody coated beads and anti-AAT Llama antibody coated beads. We also decided to use – for the first time in biomarker discovery – a real high-throughput analytical glycan profiling technique (CGE-LIF), which can be performed with broadly available instrumentation (Sanger DNA sequencer). Multiplexed CGE-LIF technology has great potential in biomarker discovery as well as product monitoring in biotechnological facilities due to the very fast analysis times, high resolution power, easy operating procedures and capability to separate N-glycan isomers. Alternative strategies include mass spectrometry using MALDI-MS (e.g. [44], [45]) or direct infusion ESI-MS, which require substantial training on the instrument and do not provide isomer separation, and UPLC with fluorescence detection (e.g. [22]), which provides isomer separation but has much lower throughput as it is not multiplexed.

The intra-batch repeatability of the strategy described in this study featured an average RSD of 16% and 20% for the AAT and IgA enriched fractions, respectively. This value includes the variation due to the protein enrichment, glycan release, labeling, purification and measurement. We have previously shown that the contribution of the measurement to this variation is rather small (6.2% [35]). The large inter-batch variation (average RSDs of approximately 33%) makes batch-corrections necessary when analyzing large sample sets.

Recent studies in human plasma have revealed that chronological age and sex influence the plasma N-glycosylation pattern [15], [16]. Likewise, our large scale enrichment of AAT and IgA from plasma samples from participants of the LLS using antibody affinity capturing followed by subsequent high-throughput N-glycan analysis, revealed that glycosylation patterns of the AAT enriched fractions are associated with chronological age and differ between females and males (Figure 3 and Table ST3 in Supporting Information S1). Interestingly, for the AAT enriched fraction, most of the glycans that are decreased with increasing chronological age are non-fucosylated sialylated compounds, while one of the fucosylated and sialylated glycans (AAT-11) is positively related to age. Levels of several oligosaccharides from the AAT enriched fractions are higher in females, as depicted by their negative correlation with sex. Two triantennary structures with fucose are higher in males, while the non-fucosylated triantennary structures are more abundant in females.

Glycosylation profiles of the IgA enriched fractions, on the other hand, hardly showed any chronological-age related alterations. Only a mono-sialylated biantennary glycan (IgA-11) could be shown to increase with increasing chronological age. This result is surprising, as it is well known that the level of galactosylated glycans on the most abundant immunoglobulin IgG decreases with chronological age [21], [22], [41], [46], [47]. Levels of several di-, mono- and non-sialylated biantennary glycans with or without bisecting GlcNAc in the IgA enriched fractions were observed to be lower in males than in females. Similarly, for IgG several glycoforms were observed at lower levels in male individuals [21].

Various differences between offspring of longlived siblings and age-matched partners of the offspring have so far been reported. It could be observed that the offspring has a lower prevalence of myocardial infarction, hypertension and diabetes mellitus [48]. In the non-diabetic individuals, non-fasted serum glucose levels were decreased in the offspring [49]. Moreover, in the offspring, larger LDL-cholesterol particles were reported [50]. We recently reported the association of two whole serum glycosylation features [15] as well as serum IgG glycoforms [21] with familial longevity. Here, we did not find an association of glycosylation features from AAT and IgA enriched fractions with familial longevity.

However, further analysis revealed an association of several glycans in the AAT enriched fractions with parameters marking cardiovascular, metabolic and inflammatory diseases (Figure 4, Tables ST5 and ST6 in Supporting Information S1). Two triantennary structures (AAT-2 and AAT-9) are positively related to both cholesterol and triglyceride levels, but negatively associated with CRP levels. Several biantennary glycans (AAT-4, AAT-6 and AAT-13) were negatively associated with BMI, CRP, triglyceride, glucose and insulin levels, but positively associated with HDL. Other glycans (AAT-1, AAT-5, AAT-7 and AAT-14 were positively associated with BMI, CRP, triglyceride, glucose and insulin levels, but negatively related to HDL. This confirms the negative relation of HDL levels with cardiovascular, metabolic and inflammatory diseases relative to BMI, CRP, triglyceride, glucose and insulin levels. Furthermore, it indicates a relation between the glycosylation of the liver-derived protein AAT with such diseases. While a relation of IgA glycosylation with disease parameters was also investigated, no associations were observed, indicating that B-cell derived proteins are less affected by metabolic diseases than liver cell derived proteins.

The proteomics analysis of the affinity-enriched IgA and AAT samples indicated a good purity of the samples. Using SDS-PAGE of IgA and AAT enriched fractions, it was demonstrated that the target proteins represent the most strongly stained components, but there are also some co-purified species, which are highly likely proteins that are bound to the protein of interest (either AAT or IgA). In-solution proteomics revealed the presence of some additional glycoproteins that would contribute to the N-glycosylation patterns obtained in this study. However, as these additional glycoproteins appeared to be of low abundance, their contribution to the observed glycosylation profiles of the enriched fractions is expected to be minor. This is in line with the fact that the observed glycosylation of IgA and AAT enriched fractions is largely in line with literature (see Tables 1 and 2). While the immunocapturing approach described here is suitable for enrichment and purification of medium to high abundant glycoproteins, other platforms will have to be considered for glycoproteins that occur at even lower abundances in complex mixtures such as human plasma. Such platforms will need to further reduce aspecific binding by minimizing contact surfaces in order to allow efficient affinity-enrichment.

The roles of glycosylation, and particularly N-glycosylation on specific proteins are not very well understood. So far, the functions of the N-glycans on immunoglobulin G are most studied and best understood. However, it may be expected that glycosylation of other plasma proteins is similarly important. In support of this hypothesis, levels of sialylation on EPO influence the proteins activity and half life [51]. Moreover, protein clearance from the blood is mediated by the asialoglycoprotein receptor, which has affinity for terminal β-galactose residues [52].

In our large-scale cross-sectional study various associations were found – similar to IgG – which indicate that actually also these two plasma proteins – IgA and AAT – show a highly regulated glycosylation that is influenced by and/or influencing various physiological parameters. Therefore, our data provide very strong support for the complex regulation of plasma protein glycosylation. Of particular relevance are the differences observed between IgG and IgA glycosylation: while the glycosylation profiles are qualitatively very similar (vast overlap of observed glycan species), there is a huge quantitative difference, with mainly a much lower degree of sialylation for IgG (Fig. SF3 in Supporting Information S1). Intriguingly, these two glycoproteins show very different dependencies on physiological parameters, which indicates the distinct regulation of IgA and IgG glycosylation, whilst they are both produced by plasma cells. As for IgA, it is worth mentioning that the observed CGE-LIF glycan profiles are qualitatively very similar to IgG glycan profiles, yet there are vast quantitative differences. The IgG N-glycosylation pattern has been characterized before using CGE-LIF [53], and even though many of the glycans observed on IgA can also be identified on IgG, their levels differ significantly. To illustrate this, an electropherogram of IgG as well as IgA derived N-glycans is depicted in Fig. SF2 in Supporting Information S1. Clearly, neutral glycans eluting in the latter part of the electropherogram (after 9000 dp) are of very high abundance on IgG, while only minor peaks are observed on IgA. This is in line with our proteomics analyses indicating that IgG contamination of the IgA samples is negligible. Interestingly, the neutral glycans found in the electropherogram of the IgA enriched fraction (IgA-11, IgA-14 and IgA-15) could not be related to chronological age, while the corresponding glycans of IgG were previously shown to be highly associated with chronological age [21], [22], [41], [46], [47].

More indications for the differential regulation of IgG and IgA glycosylation can be found in literature: in rheumatoid arthritis, the level of galactosylation of IgG decreases dramatically (e.g. [54]–[56]), while N-glycans in the IgA enriched fractions are hardly changed [54], [57]. However, our observation is of much larger significance, at it is not restricted to a specific disease situation, but simultaneously analyzes a broad set of important physiological parameters in relation to IgA (and AAT) glycosylation in healthy individuals.

In conclusion, it was shown that a strategy consisting of large-scale immuno-affinity capturing of proteins from human plasma using a bead-based method, coupled with high-throughput N-glycan analysis using multiplexed CGE-LIF is a powerful tool for the analysis of N-glycosylation patterns of specific glycoproteins in large studies. The strategy has successfully been applied to approximately 2400 plasma samples from the LLS demonstrating for the first time the complex regulation of plasma protein glycosylation for more typical plasma proteins such as IgA and AAT and not just for the IgG molecule with its Fc N-glycans that are only partially accessible.

## Supporting Information

Supporting Information S1 (PDF)
