# Supplementary material for: Targeted Biomarker Discovery by High Throughput Glycosylation Profiling of Human Plasma Alpha1-Antitrypsin and Immunoglobulin A
Source: PLoS One. 2013 Sep 9;8(9):e73082. doi: 10.1371/journal.pone.0073082 (PMC3767703; doi:10.1371/journal.pone.0073082)
Supplement: Supporting Information S1 — (PDF) [file pone.0073082.s001.pdf]

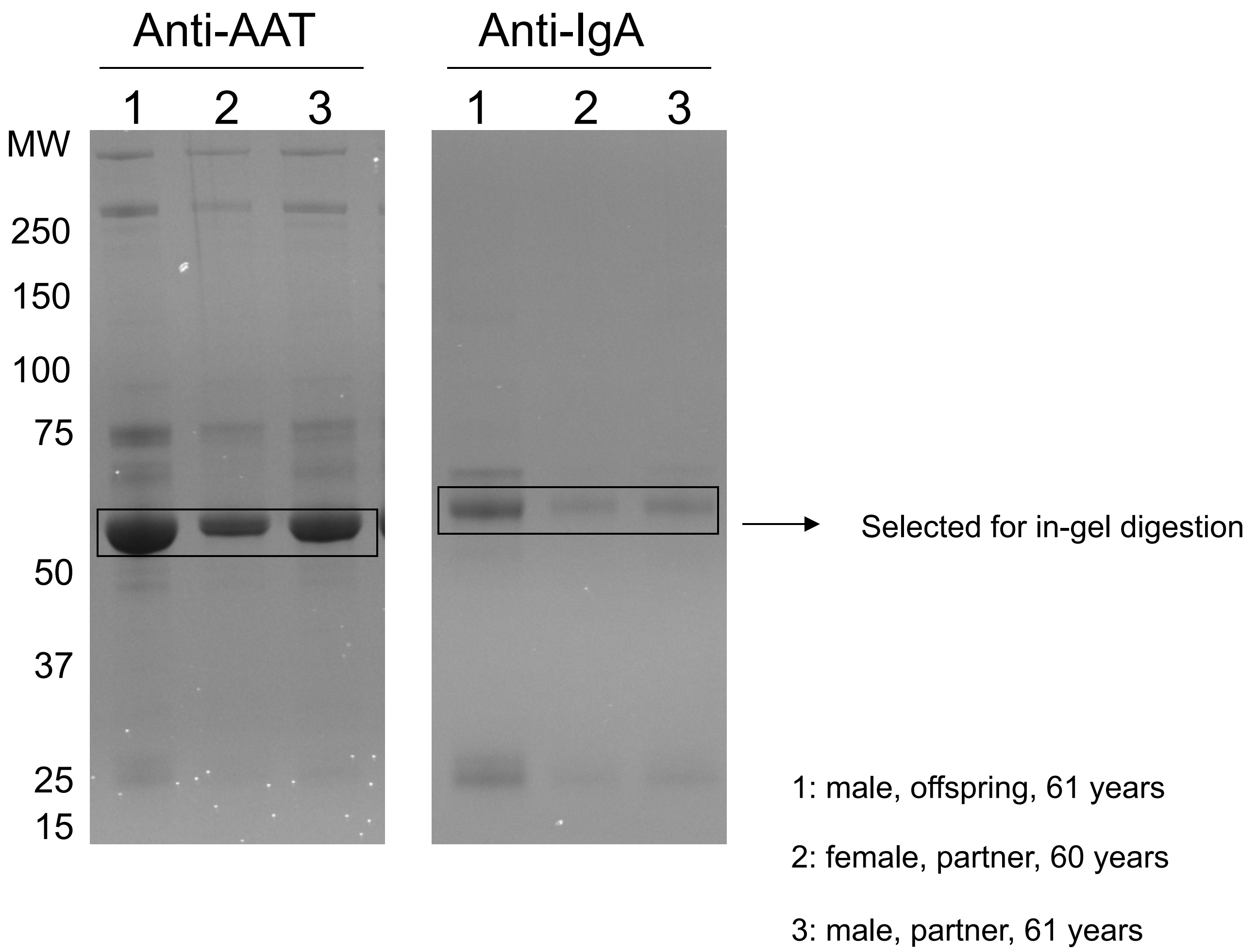

Supplemental Figure SF1: SDS-PAGE of IgA and AAT enriched fractions
Serum samples from three different subjects were enriched for IgA and AAT using antibody-coated beads and the immunocaptured proteins were analyzed by SDS-PAGE.

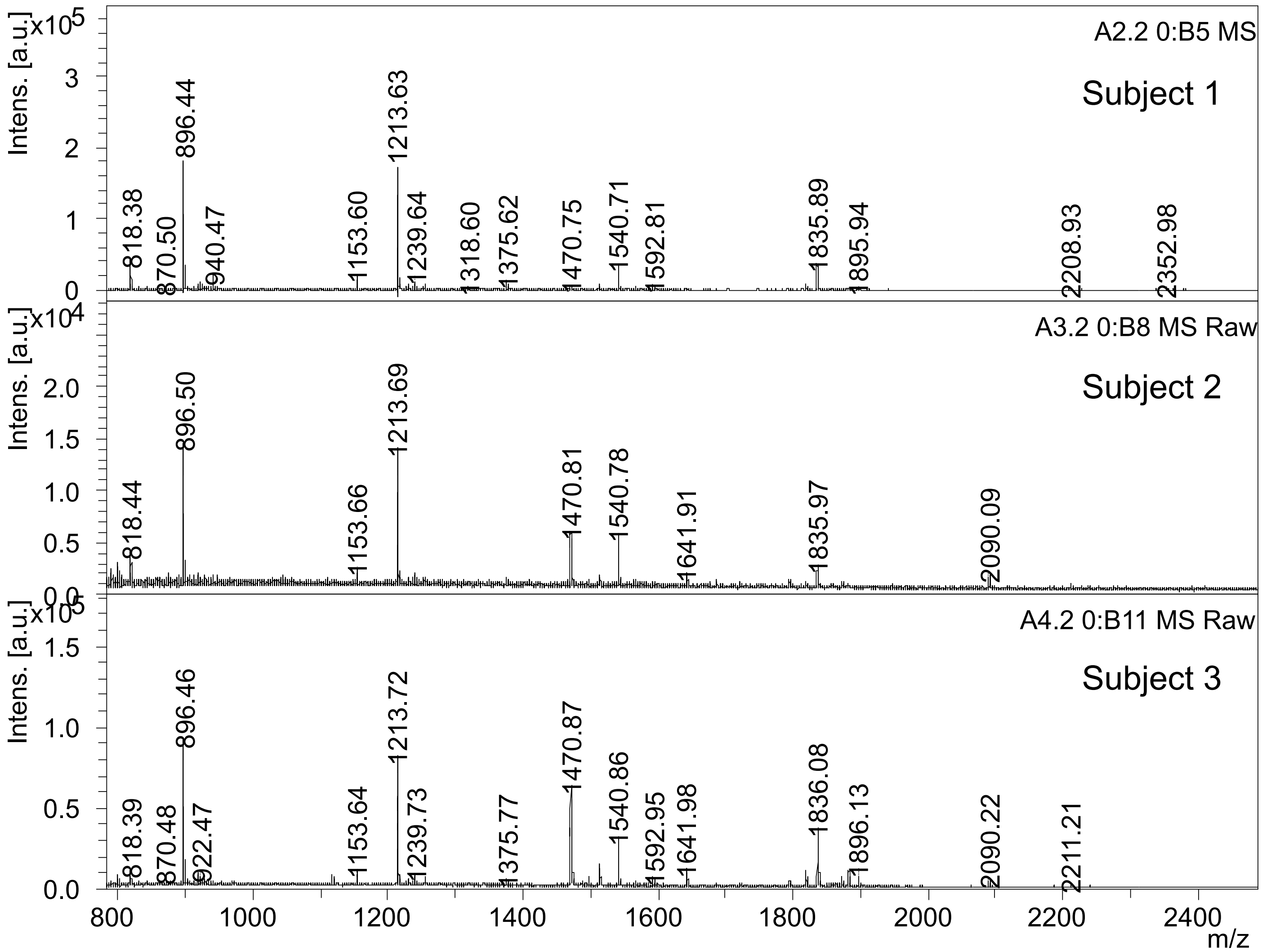

Suppl. Figure SF2A

{MATRIX SCIENCE} Mascot Search Results

Protein View

Match to: P01876 Score: 172 Expect: 1.3e-13
Ig alpha-1 chain C region OS=Homo sapiens GN=IGHA1 PE=1 SV=2

Nominal mass (M_r): 38486; Calculated pI value: 6.08
NCBI BLAST search of P01876 against nr
Unformatted sequence string for pasting into other applications

Taxonomy: Homo sapiens

Fixed modifications: Carbamidomethyl (C)
Variable modifications: Oxidation (M)
Cleavage by Trypsin: cuts C-term side of KR unless next residue is P
Number of mass values searched: 46
Number of mass values matched: 18
Sequence Coverage: 47%

Matched peptides shown in **Bold Red**

```
  1 ASPTSPKVFP LSLCSTQPDG NVVIACLVQG FFPQEPLSVT WSESGQGVTA
 51 RNFPPSQDAS GDLYTTSSQL TLPATQCLAG KSVTCHVKHY TNPSQDVTVP
101 CPVPSTPPTP SPSTPPTPSP SCCHPRLSLH RPALEDLLLG SEANLTCTLT
151 GLRDASGVTF TWTPSSGKSA VQGPPERDLC GCYSVSSVLP GCAEPWNHGK
201 TFTCTAAYPE SKTPLTATLS KSGNTFRPEV HLLPPPSEEL ALNELVTLTC
251 LARGFSPKDV LVRWLQGSQE LPREKYLTWA SRQEPSQGTT TFAVTSILRV
301 AAEDWKKGDT FSCMVGHEAL PLAFTQKTID RLAGKPTHVN VSVVMAEVDG
351 TCY
```

Start - End	Observed	Mr(expt)	Mr(calc)	ppm	Miss	Sequence	
52 - 81	3168.2517	3167.2444	3167.5081	-83	0	R.NFPPSQDASGDLYTTSSQLTLPATQCLAGK.S	
82 - 88	830.4254	829.4181	829.4116	8	0	K.SVTCHVK.H	
154 - 168	1540.7071	1539.6998	1539.7206	-13	0	R.DASGVTFTWTPSSGK.S	
169 - 177	940.4696	939.4623	939.4774	-16	0	K.SAVQGPPER.D	
178 - 200	2592.9253	2591.9181	2592.1196	-78	0	R.DLCGCYSVSSVLPGCAEPWNHGK.T	
201 - 212	1375.6151	1374.6078	1374.6126	-3	0	K.TFTCTAAYPESK.T	
213 - 221	931.5164	930.5091	930.5386	-32	0	K.TPLTATLSK.S	
264 - 273	1213.6348	1212.6275	1212.6251	2	0	R.WLQGSQELPR.E	
264 - 275	1470.7505	1469.7432	1469.7626	-13	1	R.WLQGSQELPREK.Y	
274 - 282	1153.5987	1152.5914	1152.5927	-1	1	R.EKYLTWASR.Q	
276 - 282	896.4443	895.4370	895.4552	-20	0	K.YLTWASR.Q	
283 - 299	1835.8948	1834.8875	1834.9425	-30	0	R.QEPSQGTTTFAVTSILR.V	
300 - 306	818.3771	817.3698	817.3970	-33	0	R.VAAEDWK.K	
300 - 307	946.4896	945.4824	945.4920	-10	1	R.VAAEDWKK.G	
307 - 327	2336.9819	2335.9746	2336.1293	-66	1	K.KGDTFSCMVGHEALPLAFTQK.T	
307 - 327	2352.9778	2351.9705	2352.1243	-65	1	K.KGDTFSCMVGHEALPLAFTQK.T	Oxidation (M)
308 - 327	2208.9303	2207.9230	2208.0344	-50	0	K.GDTFSCMVGHEALPLAFTQK.T	
308 - 327	2224.9220	2223.9147	2224.0293	-52	0	K.GDTFSCMVGHEALPLAFTQK.T	Oxidation (M)

Suppl. Figure SF2B

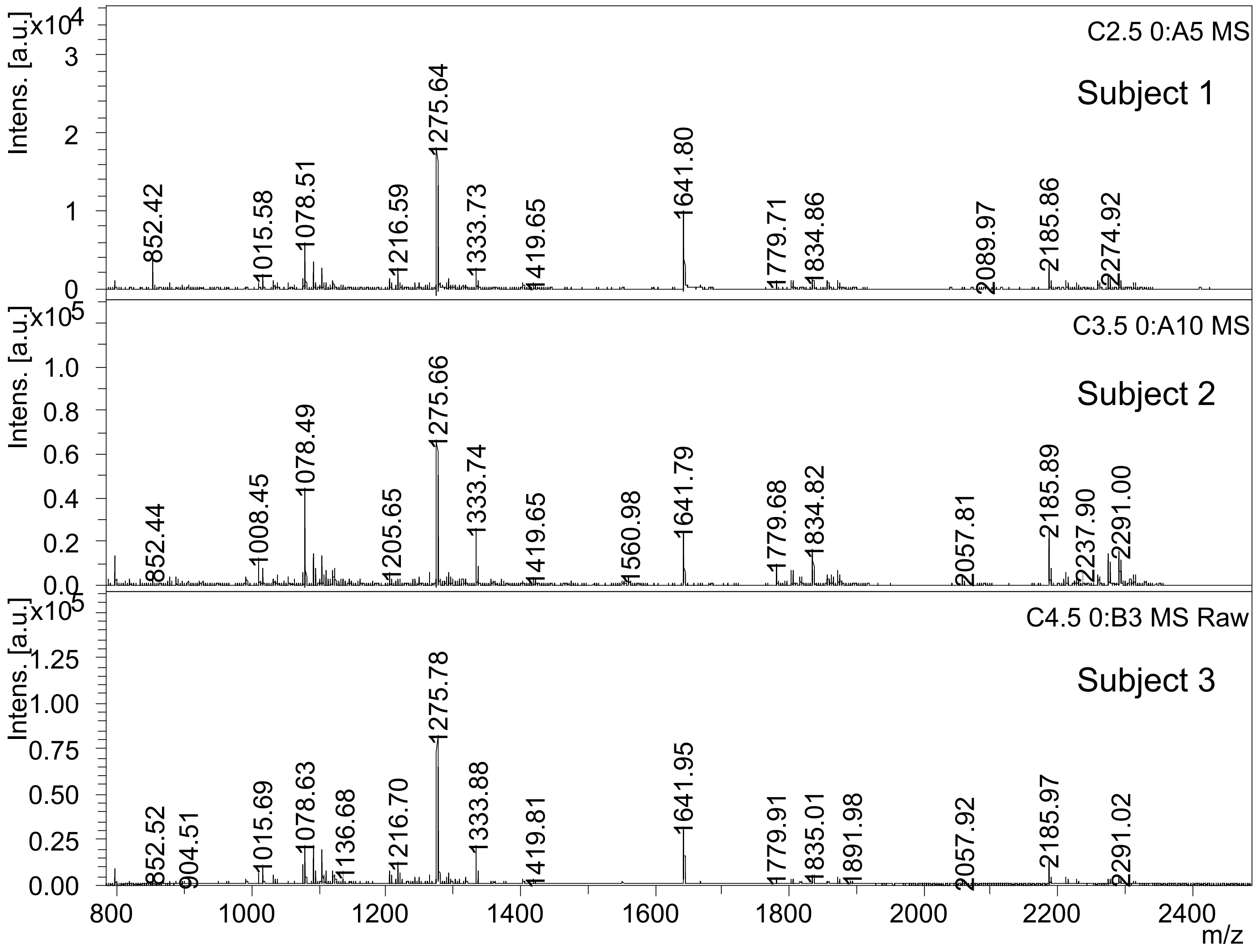

Suppl. Figure SF2C

{MATRIX SCIENCE} Mascot Search Results

Protein View

Match to: P01009 Score: 190 Expect: 2e-15
Alpha-1-antitrypsin OS=Homo sapiens GN=SERPINA1 PE=1 SV=3

Nominal mass (M_r): 46878; Calculated pI value: 5.37
NCBI BLAST search of P01009 against nr
Unformatted sequence string for pasting into other applications

Taxonomy: Homo sapiens

Fixed modifications: Carbamidomethyl (C)
Variable modifications: Oxidation (M)
Cleavage by Trypsin: cuts C-term side of KR unless next residue is P
Number of mass values searched: 60
Number of mass values matched: 25
Sequence Coverage: 47%

Matched peptides shown in **Bold Red**

```
  1 MPSSVSWGIL LLAGLCCLVP VSLAEDPQGD AAQKTDTSHH DQDHPTFNKI
 51 TPNLAEFAFS LYRQLAHQSN STNIFFSPVS IATAFAMLSL GTKADTHDEI
101 LEGLNFNLTE IPEAQIHEGF QELLRTLNQP DSQLQLTTGN GLFLSEGLKL
151 VDKFLEDVKK LYHSEAFTVN FGDTEEAKKQ INDYVEKGTQ GKIVDLVKEL
201 DRDTVFALVN YIFFKGKWER PFEVKDTEEE DFHVDQVTTV KVPMMKRLGM
251 FNIQHCKKLS SWVLLMKYLG NATAIFFLPD EGKLQHLENE LTHDIITKFL
301 ENEDRRSASL HLPKLSITGT YDLKSVLGQL GITKVFSNGA DLSGVTEEAP
351 LKLSKAVHKA VLTIDEKGTE AAGAMFLEAI PMSIPPEVKF NKPFVFLMIE
401 QNTKSPLFMG KVVNPTQK
```

Start - End	Observed	Mr(expt)	Mr(calc)	ppm	Miss	Sequence
35 - 49	1779.7076	1778.7004	1778.7609	-34	0	K.TDTSHHDQDHPTFNK.I
50 - 63	1641.8041	1640.7968	1640.8562	-36	0	K.ITPNLAEFAFSLYR.Q
154 - 160	878.4295	877.4222	877.4909	-78	1	K.FLEDVKK.L
161 - 179	2185.8574	2184.8501	2185.0327	-84	1	K.LYHSEAFTVNFGDTEEAKK.Q
180 - 187	1008.4964	1007.4892	1007.4924	-3	0	K.QINDYVEK.G
199 - 215	2089.9683	2088.9611	2089.0884	-61	1	K.ELDRDTVFALVNYIFFK.G
216 - 225	1275.6423	1274.6350	1274.6771	-33	1	K.GKWERPFEVK.D
218 - 225	1090.5281	1089.5208	1089.5607	-37	0	K.WERPFEVK.D
247 - 257	1419.6468	1418.6395	1418.6911	-36	1	K.RLGMFNIQHCK.K Oxidation (M)
248 - 257	1247.5672	1246.5600	1246.5951	-28	0	R.LGMFNIQHCK.K
248 - 257	1263.5543	1262.5470	1262.5900	-34	0	R.LGMFNIQHCK.K Oxidation (M)
258 - 267	1204.6482	1203.6409	1203.7049	-53	1	K.KLSSWVLLMK.Y
258 - 267	1220.6465	1219.6392	1219.6998	-50	1	K.KLSSWVLLMK.Y Oxidation (M)
259 - 267	1076.5674	1075.5601	1075.6100	-46	0	K.LSSWVLLMK.Y
259 - 267	1092.5604	1091.5531	1091.6049	-47	0	K.LSSWVLLMK.Y Oxidation (M)
284 - 298	1803.8988	1802.8915	1802.9526	-34	0	K.LQHLENELTHDIITK.F
299 - 306	1078.5069	1077.4996	1077.5203	-19	1	K.FLENEDRR.S
307 - 314	852.4160	851.4087	851.4865	-91	0	R.SASLHLPK.L
315 - 324	1110.5676	1109.5603	1109.5968	-33	0	K.LSITGTYDLK.S
325 - 334	1015.5799	1014.5726	1014.6073	-34	0	K.SVLGQLGITK.V
368 - 389	2258.9227	2257.9155	2258.1326	-96	0	K.GTEAAGAMFLEAIPMSIPPEVK.F
368 - 389	2274.9213	2273.9140	2274.1276	-94	0	K.GTEAAGAMFLEAIPMSIPPEVK.F Oxidation (M)
368 - 389	2290.9159	2289.9086	2290.1225	-93	0	K.GTEAAGAMFLEAIPMSIPPEVK.F 2 Oxidation (M)
390 - 404	1855.8996	1854.8923	1854.9702	-42	0	K.FNKPFVFLMIEQNTK.S
390 - 404	1871.8891	1870.8818	1870.9651	-45	0	K.FNKPFVFLMIEQNTK.S Oxidation (M)

Suppl. Figure SF2D

Supplemental Figure SF2: MALDI-ToF-MS analysis and peptide mass fingerprint results from the tryptic digest of the major bands after SDS-PAGE of affinity-purified AAT and IgA samples.

A: MALDI-ToF-MS spectrum from the major band after SDS-PAGE of IgA enriched fraction from three subjects from the study cohort

B: Peptide mass fingerprint search results from the major band of the IgA enriched sample from subject 1. Mascot score 172.

C: MALDI-ToF-MS spectrum from the major band after SDS-PAGE of AAT enriched fraction from three subjects from the study cohort

D: Peptide mass fingerprint search results from the major band of the AAT-enriched sample from subject 1. Mascot score 190.

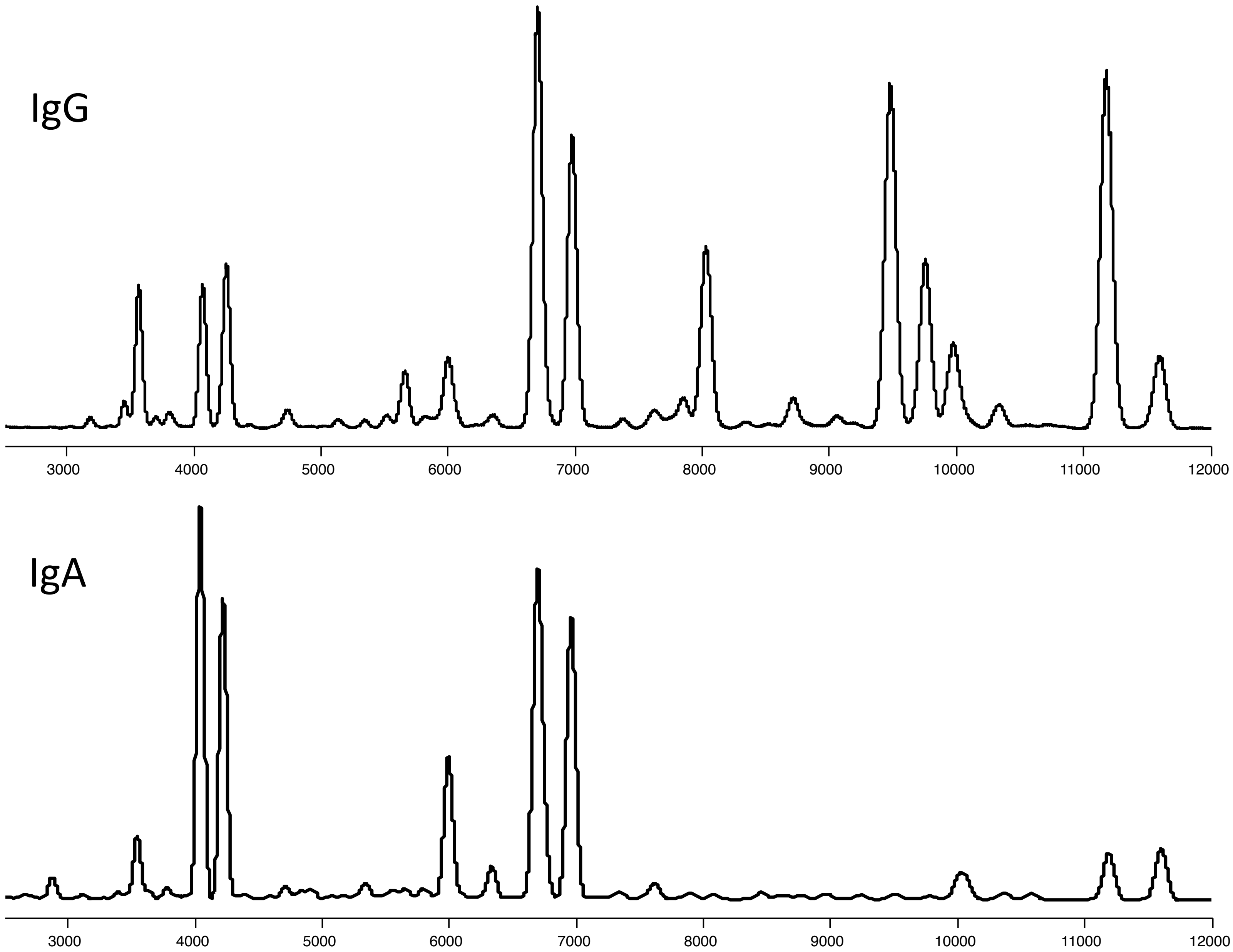

Supplemental Figure SF3. Electropherograms of APTS labeled N-glycans released from IgG (upper panel) and IgA (lower panel). Proteins were immunocaptured from the same plasma sample.

Supplemental Table ST1: Protein identification of the anti-IgA purified material after in-solution tryptic digestion followed by LC-iontrap MS/MS .
Only peptides with a score above 30 were included and a minimum of 2 peptides per protein was required.
Cover: total protein coverage observed using only peptides with score above 30. Pep total: total number of peptides (score >30). Pep unique: total number of unique peptides (score >30).

Subject 1

Protein_Name	Protein ID	Score	Cover	Pep total	Pep unique
Ig alpha-1 chain C region	P01876	1009	52	53	12
Serum albumin	P02768	838	54	36	27
Ig kappa chain C region	P01834	642	82	23	7
Ig lambda-2 chain C regions	P0CG05	292	65	14	4
Ig mu chain C region	P01871	154	21	8	7
Ig heavy chain V-III region BR	P01766	125	25	5	2
Ig kappa chain V-III region SIE	P01620	131	39	3	3
Ig gamma-4 chain C region	P01861	80	13	3	3
Ig heavy chain V-III region VH26	P01764	105	19	2	2
Immunoglobulin J chain	P01591	95	12	2	2

Subject 2

Protein_Name	Protein ID	Score	Cover	Pep total	Pep unique
Ig alpha-1 chain C region	P01876	360	28	20	8
Ig kappa chain C region	P01834	311	67	11	4
Serum albumin	P02768	287	16	10	8
Immunoglobulin lambda-like polypeptide 5	B9A064	98	11	5	2
Ig alpha-2 chain C region	P01877	201	12	3	2
Immunoglobulin J chain	P01591	111	12	3	2
Ig kappa chain V-I region	P01606	87	17	2	2
Ig lambda chain V-III region L	P80748	73	22	2	2

Subject 3

Protein_Name	Protein ID	Score	Cover	Pep total	Pep unique
Ig alpha-1 chain C region	P01876	657	46	33	12
Serum albumin	P02768	560	41	25	21
Ig kappa chain C region	P01834	522	82	19	7
Ig lambda-6 chain C region	P0CF74	162	46	7	3
Ig heavy chain V-III region BR	P01766	129	25	5	2
Immunoglobulin J chain	P01591	104	18	3	3
Ig alpha-2 chain C region	P01877	464	12	3	2
Ig kappa chain V-III region SIE	P01620	51	25	2	2
Keratin, type I cytoskeletal 9	P35527	78	5	2	2
Ig lambda chain V-III region L	P80748	99	31	2	2
Ig kappa chain V-II region TEW	P01617	63	33	2	2

Supplemental Table ST2: Protein identification of the anti-AAT purified material after in-solution tryptic digestion followed by LC-iontrap MS/MS .
Only peptides with a score above 30 were included and a minimum of 2 peptides per protein was required.
Cover: total protein coverage observed using only peptides with score above 30. Pep total: total number of peptides (score >30). Pep unique: total number of unique peptides (score >30).

Subject 1

Protein_Name	Protein ID	Score	Cover	Pep total	Pep unique
Alpha-1-antitrypsin	P01009	873	45	50	24
Ig mu chain C region	P01871	534	40	27	15
Serum albumin	P02768	496	33	21	19
Fibronectin	P02751	306	8	16	14
Complement C4-A	P0C0L4	374	12	14	12
Fibrinogen alpha chain	P02671	224	10	8	6
Ig lambda-2 chain C regions	P0CG05	185	71	8	5
Ig alpha-1 chain C region	P01876	220	24	8	5
Ig kappa chain C region	P01834	184	63	7	3
Fibrinogen beta chain	P02675	182	14	5	5
Fibrinogen gamma chain	P02679	127	15	5	5
Prothrombin	P00734	101	13	5	5
CD5 antigen-like	O43866	124	19	4	4
Apolipoprotein A-I	P02647	151	14	3	3
Ig gamma-2 chain C region	P01859	49	6	2	2
Apolipoprotein B-100	P04114	65	1	2	2
C4b-binding protein alpha chain	P04003	69	5	2	2

Subject 2

Protein_Name	Protein ID	Score	Cover	Pep total	Pep unique
Alpha-1-antitrypsin	P01009	1160	55	64	27
Complement C4-A	P0C0L4	454	19	23	22
Ig mu chain C region	P01871	432	37	19	12
Prothrombin	P00734	244	28	11	10
Serum albumin	P02768	255	18	9	8
Fibronectin	P02751	141	5	8	8
Ig kappa chain C region	P01834	223	50	8	4
Fibrinogen gamma chain	P02679	112	18	7	7
Fibrinogen beta chain	P02675	227	14	6	5
C4b-binding protein alpha chain	P04003	123	9	6	5
Fibrinogen alpha chain	P02671	132	7	5	4
Ig alpha-1 chain C region	P01876	105	21	4	4
CD5 antigen-like	O43866	105	16	3	3
Vitamin K-dependent protein S	P07225	72	6	3	3
Ig lambda-6 chain C region	P0CF74	111	28	3	2
Ig gamma-1 chain C region	P01857	63	7	2	2

Subject 3

Protein_Name	Protein ID	Score	Cover	Pep total	Pep unique
Alpha-1-antitrypsin	P01009	921	53	53	26
Serum albumin	P02768	645	45	26	22
Complement C4-A	P0C0L4	413	12	16	14
Ig mu chain C region	P01871	221	35	12	10
Fibronectin	P02751	180	6	11	11
Fibrinogen alpha chain	P02671	148	13	9	8
Prothrombin	P00734	167	23	8	8
Ig kappa chain C region	P01834	188	50	8	4
Fibrinogen gamma chain	P02679	107	21	7	7
Ig alpha-1 chain C region	P01876	132	19	6	4
C4b-binding protein alpha chain	P04003	135	9	6	4
Apolipoprotein B-100	P04114	110	2	5	5
Fibrinogen beta chain	P02675	143	14	4	4
Ig lambda-2 chain C regions	P0CG05	96	44	4	3
Ig gamma-1 chain C region	P01857	57	9	3	3
Apolipoprotein A-II	P02652	41	21	2	2
Apolipoprotein E	P02649	43	10	2	2
Complement C3	P01024	47	1	2	2
Apolipoprotein A-I	P02647	76	10	2	2

Supplemental Table ST3. Chronological age and sex influences AAT glycosylation. The direction of the regression coefficient is depicted together with its P-value. Significant results are highlighted in bold (P ≤ 0.002, after Bonferroni correction). No results were obtained for AAT_3, as values were normalized to this glycan. [a] Female = 0, Male = 1; CI, confidence interval.

	Age		*Sex*[a]	
	Coefficient x1000 (95% CI)	P	Coefficient x1000 (95% CI)	P
AAT_1	3.2 (2.2 / 4.3)	**<0.001**	-57 (-70 / -43)	**<0.001**
AAT_2	-4.8 (-6.9 / -2.8)	**<0.001**	-192 (-217 / -167)	**<0.001**
AAT_4	-6.7 (-8.2 / -5.1)	**<0.001**	-85 (-107 / -64)	**<0.001**
AAT_5	2.4 (0.8 / 4.1)	0.004	-42 (-64 /-19)	**<0.001**
AAT_6	1.7 (-0.1 / 3.4)	0.058	139 (117 / 161)	**<0.001**
AAT_7	1.0 (-0.4 / 2.4)	0.172	-102 (-121 / -82)	**<0.001**
AAT_8	-3.0 (-4.8 / -1.1)	**0.002**	-174 (-197 / -150)	**<0.001**
AAT_9	-9.4 (-12.7 / -6.1)	**<0.001**	-299 (-341 / -257)	**<0.001**
AAT_10	1.4 (0.0 / 2.8)	0.056	46 (28 / 65)	**<0.001**
AAT_11	2.7 (1.4 / 4.0)	**<0.001**	98 (81 / 114)	**<0.001**
AAT_12	-2.1 (-3.0 / -1.1)	**<0.001**	-11 (-25 / 3)	0.129
AAT_13	-7.0 (-8.9 / -5.2)	**<0.001**	-50 (-76 / -24)	**<0.001**
AAT_14	-1.3 (-2.8 / 0.3)	0.104	2 (-18 / 22)	0.854
AAT_15	-4.5 (-6.8 / -2.2)	**<0.001**	-66 (-96 / -36)	**<0.001**
AAT_16	-3.6 (-5.8 / -1.3)	**0.002**	-165 (-193 / -137)	**<0.001**
AAT_17	3.7 (1.2 / 6.2)	0.004	-139 (-172 / -106)	**<0.001**
AAT_18	-2.0 (-2.9 / -1.1)	**<0.001**	-64 (-75 / -53)	**<0.001**
AAT_19	2.8 (0.2 / 5.4)	0.032	187 (154 / 220)	**<0.001**
AAT_20	6.6 (4.3 / 8.9)	**<0.001**	057 (26 / 88)	**<0.001**
AAT_21	-2.5 (-4.5 / -0.4)	0.017	-26 (-54 / 3)	0.075
AAT_22	-8.5 (-11.3 / -5.6)	**<0.001**	-7 (-43 / 28)	0.682

Supplemental Table ST4. Chronological age and is associated with IgA glycosylation. The direction of the regression coefficient is depicted together with its P-value. Significant results are highlighted in bold ($P \leq 0.003$, after Bonferroni correction). No results were obtained for IgA_5, as values were normalized to this glycan. [a] Female = 0, Male = 1

	Age		*Sex*[a]	
	Coefficient x1000 (95% CI)	P	Coefficient x1000 (95% CI)	P
IgA_1	-2.3 (-5.8 / 1.2)	0.194	-219 (-266 / -172)	**<0.001**
IgA _2	1.5 (-1.0 / 4.0)	0.241	-68 (-104 / -33)	**<0.001**
IgA _3	4.1 (0.4 / 7.8)	0.028	49 (2 / 96)	0.040
IgA _4	-1.4 (-3.8 / 1.0)	0.254	-72 (-105 / -39)	**<0.001**
IgA _6	1.9 (0.2 / 3.6)	0.028	-90 (-112 / -68)	**<0.001**
IgA _7	2.1 (0.3 / 3.8)	0.021	-40 (-65 / -15)	**0.002**
IgA _8	1.1 (-1.1 / 3.4)	0.331	-79 (-111 / -46)	**<0.001**
IgA _9	0.8 (-0.1 / 1.7)	0.101	-5 (-16 / 7)	0.424
IgA _10	1.8 (-0.2 / 3.7)	0.073	-91 (-116 / -66)	**<0.001**
IgA _11	4.3 (2.3 / 6.4)	**<0.001**	-46 (-74 / -19)	**<0.001**
IgA _12	5.4 (3.1 / 7.8)	**<0.001**	-73 (-103 / -44)	**<0.001**
IgA _13	0.9 (-1.9 / 3.7)	0.518	-120 (-159 / -80)	**<0.001**
IgA _14	-0.8 (-2.5 / 0.9)	0.362	0,000 (-23 / 23)	0.982
IgA _15	-2.3	0.044	-0,219	**<0.001**

Supplemental Table ST5. AAT-glycosylation is associated with several biological parameters. The direction of the regression coefficient (Coef) is depicted together with its P-value. Significant results are highlighted in bold (P ≤ 0.002. after Bonferroni correction). No results were obtained for AAT_3, as values were normalized to this glycan.

	BMI		*Cholesterol*		*HDL-cholesterol*		*LDL-cholesterol*		*Triglycerides*		*Glucose*		*Insulin*	
	Coef	*P*	*Coef*	*P*	*Coef*	*P*	*Coef*	*P*	*Coef*	*P*	*Coef*	*P*	*Coef*	*P*
AAT_1	+	**<0.001**	+	0.459	-	**<0.001**	-	0.779	+	**<0.001**	+	**<0.001**	+	**<0.001**
AAT_2	+	0.511	+	**<0.001**	+	0.755	+	0.037	+	**<0.001**	-	0.313	-	0.151
AAT_3														
AAT_4	-	**<0.001**	+	0.056	+	**<0.001**	+	0.132	-	**<0.001**	-	**<0.001**	-	**<0.001**
AAT_5	+	**<0.001**	+	0.347	-	**<0.001**	+	0.344	+	**<0.001**	+	**0.002**	+	**<0.001**
AAT_6	-	**0.002**	-	0.021	+	0.005	-	0.370	-	**<0.001**	-	0.703	-	**<0.001**
AAT_7	+	**<0.001**	+	0.059	-	**<0.001**	+	0.216	+	**<0.001**	+	0.035	+	**<0.001**
AAT_8	+	0.005	+	0.007	-	0.016	+	0.235	+	**<0.001**	-	0.821	+	0.063
AAT_9	+	0.886	+	**0.001**	+	0.880	+	0.033	+	**<0.001**	-	0.023	-	0.092
AAT_10	+	0.862	-	0.745	-	0.820	-	0.791	-	0.768	+	0.115	+	0.493
AAT_11	+	0.877	-	0.231	+	0.949	-	0.503	-	**0.002**	+	0.057	+	0.298
AAT_12	-	0.098	+	0.942	+	0.707	+	0.720	+	0.945	-	0.202	-	0.706
AAT_13	-	**<0.001**	+	0.190	+	**<0.001**	+	0.158	-	0.052	-	**<0.001**	-	**<0.001**
AAT_14	+	**<0.001**	-	0.837	-	**<0.001**	+	0.767	+	**0.001**	+	0.018	+	**<0.001**
AAT_15	+	0.959	+	0.773	-	0.336	+	0.479	+	0.511	+	0.955	-	0.067
AAT_16	+	0.127	+	0.013	-	0.086	+	0.074	+	**<0.001**	-	0.635	-	0.840
AAT_17	+	0.089	+	0.113	-	0.022	+	0.211	+	**<0.001**	-	0.453	+	0.950
AAT_18	-	0.350	+	0.010	+	0.549	+	0.150	+	**<0.001**	+	0.874	-	0.142
AAT_19	-	0.827	-	0.287	+	0.820	-	0.753	-	**0.002**	+	0.780	-	0.525
AAT_20	+	0.149	-	0.530	-	0.102	+	0.788	-	0.511	+	0.301	+	0.631
AAT_21	-	0.359	+	0.529	-	0.962	+	0.269	+	0.526	-	0.318	-	0.664
AAT_22	+	0.059	+	0.641	-	0.054	+	0.548	+	0.006	+	0.949	-	0.883

Supplemental Table ST6 Relation of AAT-glycosylation features with the occurrence of inflammatory disease. The direction of the regression coefficient is depicted together with its P-value. Significant results are highlighted in bold (P ≤ 0.002. after Bonferroni correction). No results were obtained for AAT_3, as values were normalized to this glycan.

	CRP	
	Coefficient x1000 (95% CI)	P
AAT_1	36 (29 / 43)	**<0.001**
AAT_2	-28 (-42 / -15)	**<0.001**
AAT_4	-65 (-75 / -55)	**<0.001**
AAT_5	49 (37 / 61)	**<0.001**
AAT_6	10 (-1 / 21)	0.070
AAT_7	31 (21 / 40)	**<0.001**
AAT_8	-4 (-16 / 8)	0.471
AAT_9	-61 (-64 / -38)	**<0.001**
AAT_10	33 (25 / 42)	**<0.001**
AAT_11	30 (22 / 38)	**<0.001**
AAT_12	-9 (-16 / -3)	0.005
AAT_13	-47 (-59 / -35)	**<0.001**
AAT_14	22 (13 / 32)	**<0.001**
AAT_15	-13 (-27 / 1)	0.077
AAT_16	-19 (-33 / -4_	0.011
AAT_17	-15 (-31 / 1)	0.068
AAT_18	-11 (-17 / -6)	**<0.001**
AAT_19	39 (23 / 54)	**<0.001**
AAT_20	28 (14 / 42)	**<0.001**
AAT_21	-17 (-30 / -3)	0.015
AAT_22	-23 (-39 / -6)	0.007
